# Medical Essays, on Fever, Inflammation, Rheumatism, Diseases of the Heart, &c.

**Published:** 1829-01-01

**Authors:** 


### VIII.

**Medical Essays, on Fever, Inflammation, Rheumatism, Diseases of the Heart, &c. By *Joseph Brown*, M.D. of the Royal College of Physicians, of the Royal Medical Society of Edinburgh, and one of the Physicians to the Sunderland and Bishopwearmouth Infirmary. Octavo, pp. 309. London: Longman & Co., 1828.**

The title of "Essay," brings to most men pleasing, to some, very solemn recollections. It was under this denomination that their own first attempts at composition were elaborated; it was under the same that Addison, Johnson, and the other great moral teachers of England, delivered those precious lessons, which constitute the pride, and the peculiar characteristic of our literature; and the first attempt at periodical publication, among the physicians of Britain, was in the form of a series of Essays. Some mighty names, too, in the profession, have chosen this modest form of disclosing their views to the public: and it is but justice to say, that of all the known ways to its ear, this is the one which places the reader and the writer under the least restraint. The writer can be as full, as circumstantial, as eloquent, and impressive as he pleases:—he is encroaching on no man's time, for no man buys his book but he who thinks well of it; he is, therefore, pocketing no man's money, that has not, in some sort, a taste for his speculations, of which the tenor may always be known by his title-page, or table of contents; and the method in which he is to treat those, is understood to be so perfectly free, that if the reader obtains not a full view of the sentiments of the writer, he does the latter no injustice, should he pronounce him utterly incompetent for the task which he has undertaken. We know no other position in which the author of a medical paper may not feel inclined to say a little less, or a little more, than he ought; for he involuntarily turns his eye towards that control which, however gentle, he must see placed between him and the public, in whichever way he turns himself to approach it.

We have been led insensibly into these remarks, by observing that Dr. Brown has preferred this rather neglected method, of expounding his views on some of the most important subjects in medicine; and are inclined to say he has done right; since, without any invincible prejudice or interest towards our own calling of Journalists, though we undoubtedly consider periodicals the most proper channel for the promulgation of a newly discovered fact, or anomalous case, we are just as positive that he who has much to reason and combine upon such observations, will meet with more room, and quite as much indulgence from the public, in a volume of essays like the present. To the analysis of this we shall forthwith proceed.

In Dr. Brown's moderate volume of 309 pages are comprehended the eight following essays:—I. A prefatory essay;—II. An essay on Fever;—III. On Inflammation;—IV. On Rheumatism;—V. On Diseases of the Heart and its Investing Membrane;—VI. On Sympathetic Disorder of the Heart;—VII. On Ischuria Renalis;—VIII. On Stricture of the Sigmoid Flexure of the Colon.

The prefatory essay might, perhaps, have better been entitled, "an Essay on the present Education, Prospects, and State of the Medical Profession," and will be read with as much interest as any of the other valuable dissertations which follow. It is written with great power; and though, into several of the "*vexatæ quæstiones*" which he agitates, we have ourselves always endeavoured to infuse a spirit of peace,* nor can aver that we exactly coincide with the

* *Candida pax homines, trux decet ira feras.*—Ovid.

Doctor in all his positions, we do strongly recommend the clear views and energetic reasoning he displays on these subjects, to the perusal of our readers. We can merely afford space for his acute castigation of that motley diversity of medical bye-laws, which, though still allowed to regulate the education and interests of thousands, are not the same in any two colleges of the empire! "*Tot sententiæ, quot medici,*" is an old adage, which was ludicrously exemplified, the other day, before the Anatomical Committee of the lower house of Parliament: but Dr. Brown cannot see why the *medici* should be allowed to legislate separately at all on a point, respecting which their views seem so limited, and their personal interests so seducing.

"It is incredible that England should possess no school of medicine; yet such is the fact: but it is still more incredible, that, being thus circumstanced, she should affix a stigma upon men who cross the border or the channel to acquire that education which she will not give them. To the general merits of our 'time-honoured' universities, every Englishman is ready to pay homage, yet no one can say, that they are schools of medicine in any thing like the proper sense of the term; and it is notorious, that those who take degrees in physic there, acquire their medical lore in some one of those institutions which they affect to despise. It may be said, that there is no *injury* to the stigmatized class—that a man of merit has only to take the licence of the college, and he is as well received in our metropolis and its suburbs as if he were a fellow. If there is no injury, there is what is more grievous—insult; and insult offered to the whole world; though, from circumstances, more frequently received by Scotland than any other country. I am not indifferent to the thousand titles to pre-eminence among nations possessed by my country—

"Ταύτης τοι γενεῆς τε και αιματος εὔχομαι ειναι—

yet I wish Englishmen would be content to walk quietly through the world, and not insist upon stalking over it, unmindful whom they tread upon. Those titles to pre-eminence belong, not exclusively to England, but to the united empire, of which Scotland is certainly not the least gifted and intellectual portion.

"If, by the distinction of *castes*, the inferiority of the licentiates in talents and attainments is meant to be implied (an inferiority which is notoriously far from being established), then the case is dreadful indeed; for we must presume that His Majesty's lieges of Edinburgh, for instance, are as much the objects of his paternal solicitude as those of London or any other part of his dominions, yet are their lives intrusted to the care of graduates of their present benighted university. If that university cannot supply 'viri satis docti et *graves*,' why do not His Majesty's responsible advisers suggest the propriety of founding an institution in some portion of his northern dominions, which shall furnish men sufficiently *rueful* for all professional purposes? In conversation, a Cambridge graduate remarked to me, with equal good sense and good breeding,—for he was aware that Edinburgh had sent me forth,—that Oxford and Trinity College, Dublin, were the only schools they deigned to pray for. Was there ever so unchristian-like an omission as that of the northern universities? The enactments of the London College certainly prove, that the Scotch schools peculiarly require the prayers of the faithful.

"It were much to be desired, that some remedy could be found for these evils, insults, and the broils which result from them. All would be avoided were there one general system of medical instruction established throughout the empire, modified according to the pursuits of the two great classes into which our body is divided; a division of labour which is advantageous to the public, and which is sufficiently minute for every practical purpose. The ultimate object of the system should be the benefit of the community: its immediate one the securing to medical men the kind and degree of education best adapted to the attainment of the former. To each class a certain quantity of knowledge is requisite before they can embark beneficially or even safely in their respective duties. Then, how happens it that the educatiou which supplies this knowledge is left, as it were, to mere accident, and that it varies in every school in the empire? Common sense dictates, that it should be the same in each institution; and that its quantity should be fixed by the legislature, and not left to the discretion of corporate bodies, which their own enactments prove, differ in their estimates of the requisite amount. Though the only mode of procuring that uniformity of education to each class which is desirable is by legislative enactment, yet it is not clear whence the legislature is to derive the knowledge of the requisite quantity. The supplying it with this information involves a serious responsibility, which should not be thrown upon one or two individuals. Neither should the decision of the amount rest with any existing corporate body, which could not dictate to all others without exciting feelings better dormant. *A medical committee, assembled for the express purpose, would, perhaps, be the most eligible mode;* and upon their advice legislative enactments might be founded which should specify the branches of knowledge with which medical men are expected to be conversant, and the portion of time to be dedicated to the acquirement of each branch by the two classes of the profession." 11.

This essay occupies about 30 pages.

The second treats of fever, and is divided by the author himself into *malaria —illustrations of the action of malaria during and since* 1825*—contagion—the nature of fever—treatment.* Before dipping into these valuable articles individually, we ought to state, as our general opinion, that this essay is one of especial value. It presents the observations of a gentleman, who, whether we consider his late highly confidential positions in the medical department of the army,* or the extensive scale of practice in which he has been employed since he left that service, is well entitled to our attention in this still very obscure study. He has brought much reading, much acuteness and sagacity into the field; and, though he may now and then have expressed himself with more pleasantry than *we* may think fitting so grave a subject, yet every allowance ought to be made for variety of taste and animal spirits in matters of mere style; and we are persuaded that no man feels more properly than Dr. Brown himself, the *duty* and *dignity* of his vocation. Let us never forget—

"ODERUNT HILAREM TRISTES, TRISTEMQUE JOCOSI."

*Malaria* (bad air) is a term which, though naturally indefinite, has been pretty generally received in this country, since the publication of Chateauvieux, and discussion of the quarantine question in Parliament, under its Italian restriction to the miasm of marshy districts. The latter appellation is better and more classical; but Dr. Brown, and several other able writers of the present day, have been induced to employ, in preference, the term *malaria*, after the example of Dr. Macculloch, who has hazarded some bold positions on the subject. It is defined by Dr. Brown at p. 33.

"The existence of malaria is inferred from certain effects produced upon the human frame, and the source of which is presumed to be the reciprocal action of dead vegetable and animal matter with moisture, because the effects attributed to it are perceived only where such reciprocal action is possible, and because they are very generally more marked and malignant, in proportion as circumstances are more favourable to such action, as is exemplified in a marsh or jungle, exposed to a high temperature. Of its chemical composition we know nothing; but we can now trace its source, its effects upon animal life, and, to a certain extent, the laws which regulate its diffusion. This poison is not the *exclusive* product of *marshy* territories in the southern latitudes. An extensive plain, the surface of which is literally burnt by the sun, and presents no moisture, seems favourable to its production, provided the soil be rich and not sandy, the flatness precluding that underground draining which will naturally take place in countries of which the face is more irregular, and thus leaving the moisture and organized matter below the surface, in circumstances advantageous to that mutual re-action which generates miasma. The Spanish pro-

* See his dedication to Sir James M'Grigor—Director-General.

vince of Estremadura, and great part of the Alemtejo, of Portugal, form an immense plain, and, in Spring and Autumn, scarcely could a house be entered there, and this has been repeatedly put to the test of experiment,* in which some individual would not be found labouring under ague or remittent fever. This was the case at such distance from the river Guadiana, which intersects the plain, as to acquit its moisture of any share in their production; though the fevers were certainly more general and malignant near the marshy borders of the stream. And in climates ordinarily temperate, miasma may be generated during seasons of unusual warmth, in situations to which the term marsh is by no means applicable—provided they possess the requisites for its production—water on, or at a little distance below the surface, and dead organized matter undergoing the decomposing process by which it is converted into its original elements. Such generation will be most likely to occur, at times when the range of temperature is above the average of the climate at the corresponding season. Whether there is not some inappreciable state of the atmosphere, which favours the production of miasmata more at one season than another, may be matter of question. Some facts which have fallen under my observation, shewing that its abundance is not always in proportion to the usually assigned meteorological causes, would lead us to answer in the affirmative." 36.

So far the author agrees with Chateauvieux, Ferguson, Sydenham, and many others. With Dr. Macculloch, he allows this malaria to produce *intermittent* and *remittent fevers*, also cholera, dysentery, bilious diarrhœa, liver disease, and jaundice, but finds reason to doubt of rheumatism and neuralgia, which the same writer has added to the catalogue.† Nevertheless Dr. Brown relates the case of an officer (p. 44) affected with neuralgia facialis, which, though otherwise explicable, seems not unfavourable to that hypothesis. This case we shall here insert.

"Neuralgia has been mentioned as a product of malaria. This will appear startling to some; but as the following case seems to favour a belief in such causation, and to be consequently in some degree illustrative of the action of this poison in the district, I will detail it. Valeat quantum valere possit! An officer in the army consulted me in 1827 on account of neuralgia affecting apparently the portio dura of the seventh and second branch of the fifth pair of nerves. Severe pain occupying the left cheek, temple, and portion of the face immediately below the orbit, with copious lachrymation from the eye, and mucous discharge from the corresponding nostril, came on every morning at nine o'clock, and ceased at two, P. M., leaving merely a slight sense of soreness in the part. He was asked whether he had ever suffered in the same way before? He replied, once very many years ago, and *that* attack he attributed to cold caught on night piquet at Carrickfergus, in Ireland, during the rebellion. On my inquiring as to the nature of the soil and country about Carrickfergus, he told me it must be marshy, for he remembered being much struck with the appearance of ignis fatuus, a phenomenon he had never before observed. For his present attack (after attention to the primæ viæ) the sulphate of quinine was given with much advantage; but as, after taking it for some time, the pain, though much less severe, still continued to recur in some degree, the carbonate of iron was administered, and he became so far well as to be able to discharge his military duties, having no pain at all, but what he termed a slight uneasiness of the head. Change of air was recommended, and he went for a fortnight into Scotland, to the neighbourhood of Stirling, and returned thence in perfect health. Afterwards he informed me, that when cold and damp easterly winds prevailed, to which the barracks in which he is quartered are much exposed, being close to the sea-beach, he felt some uneasiness, but it never amounted to an actual neuralgic attack."‡ 46.

---

* By the British medical officers.

† It is curious that neither he nor Macculloch have noticed the *ulcerated legs*, so frequently observed in the West Indies, as a minor effect of *malaria*.

‡ For various other illustrations the reader is referred to our Review of Dr. Mac-

Dr. Brown has proved, beyond all controversy, that, within the last few years, say since 1825, intermittent and remittent fevers have begun to show themselves with considerable frequency, in districts of the eastern coast of England, from which they were long supposed to be finally banished. Thus at Sunderland, Shields, and Newcastle, they appear pretty frequently, and the same has been the case, as we are well informed from another source, around Hull, and in the northern parts of Lincolnshire, formerly free from this complaint. It was to prepare the reader for an explanation of this singular revolution in atmospheric influence, that we transcribed Dr. Brown's views of miasma at such length. In short, as heat and moisture are necessary to the organic decomposition during which the aguish miasm is evolved; and if draining, as is well known, has formerly succeeded in banishing ague by *lessening** the quantity of moisture necessary for that process, it follows, that years in which more moisture than usual is deposited on the ground, or in which the rays of a sun more powerful than usual penetrate, and produce decomposition, to a greater depth in the soil than formerly:—may, in either case, generate a mass of miasmatic air, if not as potent as before the land was drained, yet sufficiently so to produce ague, and the other diseases of its class, among the predisposed. We have only to peruse the medical history of any place recently dried in the above described manner, such as frequently occurs in the American medical journals, to learn how precisely this view corresponds with facts.

In favour of cholera deriving its origin from this same malaria, our author makes the following remarks.

"But the most decisive proof of cholera's being the effect of malaria is to be found in the fact, that many of the most obstinate cases of remittent fever, which have occurred in this district, commenced with that disease. During the autumn of 1827, I treated a case, which commenced with cholera, passed into remittent fever, and finally into obstinate quartan ague, ultimately cured by sulphate of quinine. I specify this case, because the gradation was striking; but during the two preceding years, the number of cases in which remittent fever commenced with cholera, was very great. Writing in October, 1825, I remarked—'Fever is at this moment prevailing, and in most of the cases the hepatic functions have been prominently affected, many of the cases began with bilious diarrhœa, many with cholera.'"† 37.

Dr. Brown considers that jaundice frequently originates from a dilute state of this malaria.

"I speak not of it, as of a concomitant or sequela, as it so frequently is of intermittent and remittent fever; but as an independent disease, at least, dependent only, as jaundice always is, on disorder of the liver, its ducts, or, perhaps we may add, of the duodenum. The British army marched from Paris into French Flanders in the beginning of 1816. Part of it was stationed in Artois, on the somewhat low and flat banks of the Scheldt. The country being well drained and cultivated, ague was rare, and remittent fever by no means prevalent; but the slower operation of malaria was visible, during the first two years the army remained there, in the extraordinary number of cases of jaundice which occurred. Many of these proved very intractable." 41.

Here he relates a very instructive case of that disease, and adds, "as our troops became accustomed to the climate, this disposition to jaundice disappeared."

---

culloch's work in the present number of the Journal. We could add many instances from our own experience, but they are unnecessary at present.—*Ed.*

* For no draining could remove the whole.

† Edinburgh Journal of Medical Science, No. 1, p. 10.

On the subject of contagion *propagating* aguish fevers, Dr. Brown brings forward the testimony of Dr. Cleghorn (Minorca, p. 132) and of Dr. John Clark, (works, vol. 1, p. 152,) and the case of a gentleman, who, on going to Darlington to fetch his son, who had been seized with ague, while at school there, became affected with the same complaint. He adds, "the common objection that to attribute a disease to two sources is an unphilosophical multiplication of causes, has really no force. It is true of the itch, for instance, that it may arise from mere filth, and yet be propagated by contact; or if a purulent discharge take place in ophthalmia, especially in crowded barracks, how rapidly does the disease spread to others from the individual in whom it was first engendered, though neither this individual, nor those successively attacked, may have laboured under any thing of the kind for years." 61. The author might further adduce the evidence of Sir John Pringle;* but we have ourselves, at other times and places, dilated so largely on this topic, that, at present, we spare our readers, although much tempted to discuss upon this occasion, the excellent inaugural essays of Drs. Thomson and M'Gee, two experienced and sensible naval surgeons, who have, within the present year, maintained with much talent the impossibility of that communication, for the *reasonableness* of which Dr. Brown offers these arguments.

The public are already in possession of Dr. Brown's views of the nature of fevers, in his essay on that subject already quoted. He contends for the general principle of a specific poison, though he is doubtful whether that poison primarily exerts it action upon the nervous system. But he powerfully combats the inflammatory origin of it, whether asserted by Broussais, Clutterbuck, or Erasistratus, and those who feel inclined to see the *coup de grace* given to the first of these theorists, will read this essay with delight. It is indeed a butterfly broken upon the wheel. But we are tempted to introduce a specimen of our author's caustic criticism.

"Let us now consider that form of the phlogistic theory which, partly from a considerable portion of talent possessed by its author, M. Broussais, and embodied in the doctrine, but still more from the zeal displayed in extolling its merits by a host of active partisans, has obtained the most ascendancy over the public mind. Regarding its matter, opinions may be divided; but the manner in which its author has unfortunately chosen to send it forth to the public is calculated to excite but one feeling, that of unmingled reprobation. The tone he assumes towards all those whose writings are not, so far as they go, in perfect parallelism with his own, is insulting in the extreme. He even avows, in the preface to the most systematic of his works, the intention of rendering their writings ridiculous.† This charitable intention is, however, seconded by powers of satire so feeble, that he has been successful in making only one doctrine ridiculous, and that unfortunately is the only doctrine in the world which he had not destined to this enviable distinction. We excuse, nay even admire, a little exuberant warmth in the honest advocate of abstract truth; but with that of M. Broussais (and he displays a great deal) there mingles an air of selfishness; for it is always excited by some notable matter, of which he claims the patent-right; and there is in it a constant reference to the 'Moi,' to use a favourite term with him, which strips his controversial writings of every semblance of respectability. There is a disingenuousness, too, about him, which throws a suspicious shadow over every thing he utters. Does a fact oppose his doctrine, it is kicked out of the way without any ceremony; for he is in the habit of distrust-

* Some of the men, during the voyages, being taken ill of the remitting fever, this fever, by the crowds and confined air on board was soon converted into the jail distemper, and became infectious."—*Diseases of the Army*, p. 39.

† Examen des Doctrines Medicales et des Systêmes de Nosologie, preface, p. 11.

ing all facts observed by 'erring and prejudiced minds (esprits faux et prévenus),' without apparently perceiving the very questionable predicament in which the extension of such a principle would place all those related by M. Broussais. 'The English have *invented* a new disease, under the name of delirium tremens,' and cures of this '*pretended* disease, obtained by repeated doses of tincture of opium,' have been published in France. 'It would be bad manners to give us the *lie*;' but he would like very much to know 'what becomes of the *victims* of similar cures,' and especially is he desirous of 'learning afterwards the state of the corpse.' Should a case of the disease occur to this writer, if he will treat it by leeches and gum-water, an opportunity will soon be afforded him of gratifying his curiosity in the latter respect.

"Such is the candour which pervades the writings of the professor of Val-de-Grace. Every thing that is not in perfect accordance with the '*doctrine physiologique*' is railed at in set terms. In general he seems to proceed upon the stoical principle of the perfect equality of all deflections from the rule of right. So long as the opinions of a writer run parallel to his own, it is very well; or, at most, a slight intimation is given, which occasionally ripens into a positive accusation, supported by some comparison of dates, of a little pilfering from the grand repository in the fauxbourg St. Jacques. But the instant deviation is perceptible, the word 'ontologie' is pronounced, and woe betides the creator of the abhorred abstraction. M. Broussais would do well to reflect that what he stigmatises by this very felicitous term, for the proprietorship of which he contends as strenuously as if it were a prize worthy of litigation, is inevitable in diseases 'incertæ sedis,' the precise nature and site of which are as yet unascertained. No man is an ontologist in ophthalmia, pleurisy, or pneumonia. With regard to those fevers considered essential, we have not attained such precise ideas; but certainly during the last quarter of a century no pains have been spared to explore every path—observation of symptoms, analogy, *post mortem* examination—which leads to a perfect knowledge of the subject. I am convinced that our acquaintance with it has increased, and I will do M. Broussais the justice to add, that he has, *in some degree*, contributed to this increase. But till we know the whole matter, till we have reached some one point (*if such exist?*) whence all the phenomena diverge, and ascertained the precise nature of the affection of that point, we must be content to take these as we find them, and do all we can for their alleviation. M. Broussais says he has found this point for us; but unfortunately he has forgot that others can be convinced only by the same means which have furnished him his conviction—the evidence of facts; and that these facts have not been published."*

Respecting that peculiar congestive action also, which has been so largely described by Dr. Armstrong, he expresses his doubts. Still, as the latter once observed to a friend of ours, "if the phenomena which present themselves in a softened spleen, for example, of a person just dead of a fever, are not the results of congestion, what are they?" You have blood accumulated in the part, the tissue broken down, altered, more brittle than a jelly; the organ is loaded with blood, but without effused blood; nor is there any one trace of inflammatory action to be perceived in the whole mass, neither redness after death, nor pain during life, nor any of those well known effects, commonly named *terminations of inflammation*. We confess we never found greater difficulty in comprehending the act of congestion, than the act of erection in the tissues of that name, to which state, indeed, it is nearly allied.

In Dr. Brown's treatment of fever, we meet with nothing particularly novel. He is less anxious for bleeding than some writers, and in the last stages, he is more liberal of wine and opium than others; and of mercury he says, "its employment in typhus is highly expedient, both as a purgative and alterative;

* The reader will find a rich treat of acute criticism between the 77th and 92d page of Dr. Brown's work.

and is still more imperiously requisite in remittent fever, where the biliary functions are so prominently affected." 120. Emetics, *very moderate* cathartics, and sulphate of quinine, in the intermitting forms, he also patronizes. One valuable caveat, for young hands, we transcribe.

"The dose in which I employ it (the sulphate of quinine) is two grains every three or four hours, during the intermission, in any agreeable distilled *water*. The astringent vegetable infusions, such as that of rose leaves, do not appear to me to be proper menstrua for it. It is certainly exceedingly insoluble in such infusions; and I suspect that this arises from some decomposing effect of the astringent principle. In intermittent I have rarely found it to fail, provided the apyrexia were perfect. But so far as my experience extends, and it has not been very inconsiderable, it does not appear to be a remedy for any stage of fever actually existing, whether remittent or continued." 124.

The third essay, upon inflammation, will not detain us long. The author himself considers it prefatory to the ensuing essays; in short, it is a profession of his creed on the subject of inflammation. He deems twenty ounces a small, and thirty ounces a large bleeding, if the patient be vigorous; and very properly protests against considering syncope as any thing else than an untoward event, to be avoided as much as possible. Antimony and colchicum he pronounces extremely useful, and delights us, by finding his extensive experience agree with that of Dr. Armstrong, on the benefit to be derived from opium in inflammations of the stomach and bowels. He narrates a case of obvious gastritis, page 153, which was undoubtedly overcome by frequent doses of the black drop. This our author prefers to every other form of opium, even to Battley's sedative liquor, except when he wishes to produce an astringent operation, which he finds best effected by solid opium, or laudanum. We register these remarks, however insulated; being sensible that a knowledge of the respective *powers* of remedies is only to be acquired from the observations of practical men.

Essay fourth is on rheumatism, he defines it "to be inflammation of the muscular and fibrous structures." Bloodletting in rheumatism he holds eminently dangerous.

"We cannot attempt to cure rheumatism by bleeding and other evacuants, without imminent risk of causing metastasis (perhaps fatal) of the disease to an important internal organ, most commonly to the heart, its investing membrane, or both. It is true that we may treat the disease as cautiously as possible—however prudent we may be in the employment of evacuants, or should we abstain from them altogether—yet will its tendency to affect the heart be frequently found very decided: but, I must say, that all the cases of speedy fatality that I have observed, have occurred after copious depletion." 158.

The pericardium is the *principal* seat of this secondary and fatal affection, and Dr. Brown has found that the inflammation of the heart which occurs without a true metastasis, the disease still remaining in the joints, and which has taken place "where depletory measures have not been at all, or moderately employed, has either been entirely subdued by the means adopted, or has terminated in hypertrophy, or other chronic forms of cardiac disorder, requiring the adoption of the means which will be explained in the next (V.) essay." Nevertheless, when the heart, or its investing membrane becomes affected, whether the disease still exist in the joints, or there be total metastasis, *we have no refuge but in those decided depletory measures which are so much deprecated by Dr. Brown, in the first attack.* From the great attention Dr. Brown has paid to the subject; from his ample experience, and convincing cases; from the prevalence, finally, of organic disease among the almost subterranean population around him, we would entreat the attention of our readers to this fatal metastasis. The following *rationale* is interesting. See page 159.

"The muscular structure of the heart, and the fibrous nature of its investing membrane, furnish the only explanation I have to offer of the great tendency of the disease to assail or to be translated to these organs. If Dr. Godman's views be admitted, and they appear to be the result of very accurate examination, there is no difficulty regarding the pericardium; for it is *positively continuous with portions of the fibrous texture which is the seat of external rheumatism.* 'The layer,' says he, 'of the fascia superficialis, immediately covering the thyroid gland (described as the fifth process, p. 34) passes under the sternum to the surface of the arteria innominata, where it is joined by the outermost layer of this fascia, covering the lateral and back parts of the neck. Over the subclavian artery; from its inferior edge the fascia extends outwards and downwards.—We then trace the fifth process, or thyroid layer, (in union with the outer part) of the fascia superficialis down to that part of the arch of the aorta where the serous membrane of the pericardium is reflected to form the immediate covering of the heart. The serous membrane being cut through, we can raise the fascia from the surface of the aorta, down to the commencement of the fleshy fibres of the heart, with as much ease as we can elevate the outer or floating portion. However singular it may appear, that this arrangement should not have been discovered until this time, it is by no means so singular, as that anatomists during so long a time should have continued to believe that a *serous* membrane, like the pleura, could form a strong fibrous membrane, like the pericardium.' "*

One moderate bleeding is all that Dr. Brown ventures upon in rheumatism, for the important reason already referred to, and colchicum and opiate diaphoretics complete the cure.

To the fifth essay, of 107 pages, on diseases of the heart and its investing membrane, it is impossible to do justice in the present sketch. It constitutes, in short, a complete treatise on the subject, and contains several excellent cases and dissections, in confirmation of the author's views; which are enlarged and novel, whether we consider the causes, the symptoms, the pathology, or the cure of these, till lately, ill understood, or neglected maladies. Their appearance, he suspects, is at present of much more frequent occurrence than heretofore; and the exciting cause which he places at the head of his catalogue, is rheumatism, an observation in which he was surprised to find himself supported by Scudamore. *Excessive motion, atmospheric impurities, atmospheric vicissitudes, abuse of spirituous and fermented liquors*, which is, both directly and mediately, a frequent cause of affection of the breath: *disease of the lungs*, and *congenital disproportion*, constitute other causes which he names. He adds a curious example of the effect of *moral feelings*, p. 175.

In passing in this summary manner to the next essay, we would not have it suspected that we think lightly of the present part of his work. On the contrary, we beg leave to assure our readers, that he has treated the whole as a master, and that the essay abounds every where with original remarks; but, as these are quite too numerous for being extracted, and we should not wish to deprive the reader of the pleasure of perusing the treatise under that elegant synoptical form in which it is presented by the author, we hope to be excused from transcription, where to transcribe would be to mangle and dismember. We shall content ourselves with one interesting extract.

"The following was, I think, a case of neuralgia of the heart:—

* Anat. Invest. p. 34.

"A gentleman advanced in life requested my attendance on the 5th of December, 1826. The account I received of his symptoms was as follows: He had for some time found any exertion beyond a slow walk on level ground distressing to him, by inducing a sense of uneasiness under the sternum, which he felt great difficulty in explaining; the most correct idea he could convey of it was by comparing it to the numb feeling of the leg or arm induced by pressure on their nerves. The sensation was such as to compel him to rest midway in a very gentle ascent of two or three hundred yards, which his business obliged him to ascend daily. This state of things had existed many months; but the feeling on account of which I was summoned, was of but a few days' duration: this was an agonizing pain felt within the whole of the precordial region, from the lower part of the sternum to beneath the left mamma, and which diffused itself thence over great part of the thorax, and down the *left* arm to the very points of the fingers. It was also felt severely in the shoulder, and still more so in the ulnar nerve at the elbow. *This pain occurred daily at six, p. m. and continued till the morning without any abatement; it then spontaneously subsided, to be renewed in its former intensity after an interval of about nine hours;* but exertion of any kind, such as ascending the staircase, would bring on the paroxysm even in the forenoon. During the intensity of pain, the action of the heart was feeble; and the countenance was pale, and expressive of great anguish. The stethoscope did not indicate any thing, either during the pain, or when it was absent, but a degree of dulness in the sound of the ventricle.

"He had been bled the day before I visited him, by his surgeon. A few additional ounces of blood were drawn from the arm, which exhibited no buffy coat, and six grains of compound calomel pill, and a draught with thirty drops of tincture of opium, were given.

"Dec. 6th.—The pain did not subside before the usual time, being apparently uninfluenced by the measures adopted. Pills of calomel and colocynth were administered, followed by some castor oil; and, in the event of the pain recurring, he was directed to take a draught containing twenty drops of the black drop, every two hours, till it should be subdued, and to continue the compound calomel pill.

"Dec. 7th.—The second draught gave entire relief; the pain, instead of remaining fifteen hours, having been subdued in two.

"The compound calomel pill, the opiate, and an occasional laxative, were continued for a few days, and the neuralgic affection entirely ceased; but those obscure feelings which he had previously experienced on exertion, especially on ascending a hill, still remain."* 221.

In the next essay, Dr. B. has grappled with that difficult subject, the sympathetic affections of the heart. When there is palpitation, intermittence of action, pain in the region of the heart, and occasionally a tendency to syncope —then there are so many indications of this state present. "There are two circumstances to be considered in palpitation: the actual movement of the heart, and the individual's perception of it. The action of the heart, like that of other internal organs, when perfectly healthy, proceeds, unperceived by its owner; but, in sensitive states of mind, *the patient may be conscious of deviations from the healthy character, very slight in degree.* I have repeatedly found dyspeptic and hysterical patients complaining of distressing palpitations, when, in truth, the heart's action explored by the stethoscope, though found to be frequent, forcible, or irregular, was by no means very considerably so." 275.

*Sympathetic* intermission of pulse, he says, is in nothing more remarkable than the *patient's immediate perception of it.* If the heart miss one stroke in a minute, the individual will exclaim, "now my pulse is intermitting." We ourselves know an instance of this kind in an eminent physician, whom, were

* If the foregoing case be considered in connexion with the observations made in the review of Dr. Macculloch's work, in the present number of the Journal, we think the reader will have little difficulty in concluding that it was one of *intermittent* neuralgia, and consequently, connected with the tribe of intermittents generally.—*Ed.*

it possible, we should believe Dr. Brown is here describing. He subjoins several weighty reasons for conceiving that the affection named angina pectoris, "is a spasm of the heart depending frequently on an irritation of distant parts, as the brain, or digestive canal." 281. He ends, however, with an encomium upon Harrowgate water, which we do consider overcharged. Dr. B. felt himself benefited by it—granted: and so did Lord Gardenstone find himself better from St. Bernard's Well, and in gratitude, adorned it with a statue of Hygeia:—but has any one since ever had reason to be so grateful? Truth is, that just as some men grow sick and die, others grow sick and then get well again, whether they keep the bowels open by fetid mineral water, or by other contrivances; and we apprehend the *post hoc, ergo propter hoc*, has misled our very accurate reasoner for once, in the present instance. He attributes this power of Harrowgate chiefly to the muriates of soda, lime, and magnesia, which certainly are salts contained in it, in great abundance.

The seventh essay presents us with several valuable, and well detailed cases of ischuria renalis. In neither of the two longest was there seen any thing confirmatory of calculus being the exciting cause; in both, there was topical pain, and in the one which proved fatal, indubitable tokens of inflammation of the kidney were seen on dissection. From this, and other considerations which he sums up in a skilful manner, Dr. Brown concludes, that suppression of the urinary secretion, in other words, the essence of ischuria renalis, depends most frequently upon inflammation of that organ. He adds,—" I will venture to suggest, that ischuria renalis should be treated on the ordinary principles applicable to inflammation, modified in degree, according to the constitutions of the individuals who are usually the subjects of the disease—men whose stamina are impaired by gout and gravel, or by the habits which usually engender these maladies." 296.

He recommends mercury after due depletion, and, as this mode of cure will not at all interfere with the antispasmodic practice usually adopted, and often not very perseveringly pursued, we consider his suggestion as being, upon the whole, an improvement.*

The eighth and last dissertation, we have said, treats of stricture of the sigmoid flexure of the colon. "So many cases of it," he says, p. 298, "have fallen under my observation, that *I think I can always recognize it*, and hope to be able to state the indications by which its existence will be rendered equally perceptible to others; a point which may be found not entirely devoid of utility." He explains at length the true nature of this formidable affection, as confirmed by what he has seen in the dead body, and then proceeds to detail the history of the symptoms, in that order of succession in which they usually appear.

"At first there is difficulty, but that not very considerable, of regulating the bowels; with occasionally a slight feeling of uneasiness and aching in the left iliac region, extending thence a little below the umbilicus, towards the ileum of the opposite side. This difficulty, however, goes on increasing; purgatives are consequently varied in kind and increased in strength; but though evacuations be obtained daily they are small, liquid, and unsatisfactory. With the augmented difficulty of regulating the bowels, there is an increase of the feeling of distention, with flatulency, furred tongue, and inappetency. At length, the purgatives cease to act, and then there is distressing pain of the abdomen, which becomes extremely distended. This distention can be distinctly traced from the left flank, running across the upper part of the abdomen, immediately under the stomach, to the ileum of the opposite side, and in this direction the pain is now experienced. The explanation of the changed direction of the pain—for previously it crossed the abdomen below the umbilicus—

* Sketches of one or two of the cases of ischuria will be found in the Periscope.

seems to be, that in the ordinary state of the colon, this intestine lies in the cavity with the convexity of its curve downwards; whilst in the distended state its position is reversed, the middle of its convexity being opposite to the right extremity of the great curvature of the stomach, and nearly in contact with it. Though the smaller intestines are distended, yet the greater diameter of the colon enables us to trace its semicircle very correctly across the abdomen. The distention, too, is very perceptible in the cæcum, and the pain is often felt severely there. From the extreme obstinacy of the bowels, the medical attendant is, perhaps, led to examine the rectum with a bougie, though there has been no feeling indicative of disease of that intestine, but he finds it perfectly free from obstruction. Vomiting now takes place; and probably for a couple of days every thing taken into the stomach, and even the contents of the bowels are discharged in this way. The patient rarely, indeed, succumbs to this first distressful paroxysm; a calm generally ensues; purgatives resume their power over the bowels; the tension of the abdomen is diminished; and he is restored to *comparative* health. But his tongue will generally be found white and coated, or perhaps white at the edges, and red and glazed in the middle; and there is still uneasiness in the abdomen now experienced in the original situation, that is, below the umbilicus. After this calm has endured an uncertain period, perhaps from a month to seven weeks, accumulation again occurs, with a renewal of all the violent symptoms. This alternation of comparative calm and paroxysms of intense pain and vomiting may exist for a considerable length of time, even for a year, or double that period; but ultimately existence is terminated, most commonly under the agonizing circumstances mentioned, the bowels giving way at one point, and their contents being effused into the peritoneal cavity; though occasionally, as has already been remarked, the patient sinks previously to the occurrence of rupture and effusion.

"This sketch is drawn from cases of the disease which I have had an opportunity of watching for a considerable length of time—even for years; but when called, as I have occasionally been, towards the fatal close, I have generally been able to gather a history of gradually increasing constipation and of spontaneous efforts for relief by vomiting, considerably resembling those which have been described. Should even the narration of symptoms be less distinct, yet if we find unyielding costiveness which has continued long, and gradually increased, without the indications of enteritis, or stricture of the rectum; if at the same time we can trace accumulation along the great intestines; and if the patient, even when he thought himself in comparative health, has experienced a degree of permanent, though slight uneasiness, extending from the left ileum towards that of the opposite side, we need have little doubt of this being the form of disease under which we find him labouring." 303.

In this always fatal affection, our author assures us that the stricture is found at the sigmoid flexure of the colon, varying in length from one inch to four, and often so much contracted as scarcely to admit a *crow-quill*, its sides hardened, thickened, and confused, by the infiltration of coagulable lymph. Bleeding, and especially mercury, frequently produce an alleviation, but Dr. B. has not seen a cure from either of them. A very instructive case of intus-susception, and another of adhesive strangulation, is given at the close of this essay.

The author is a powerful writer, an original and resolute thinker:—and, favoured, as we learn he has been all his life, with overflowing opportunities for medical observation, it is still more gratifying to be able to add, that he seems to ingraft the results of that observation upon a large and well-chosen stock of acquired professional knowledge. His eloquent style also bespeaks the man of general and tasteful reading; although we sometimes meet with inequalities in it, that could never have caught his ear to the southward of the Humber. The few defects in his arguments which we observed, have been noted sufficiently, and with these abatements we dismiss him, after many thanks for the pleasure and instruction he has afforded us, and with the hope that he will not allow the enviable talents he possesses to remain inactive.

